# Comparison of diagnostic performance between the World Health Organization and the Centers for Disease Control and Prevention real-time PCR assays for molecular detection of the mpox virus

**DOI:** 10.1128/spectrum.01431-26

**Published:** 2026-08-14

**Authors:** Darwin Paredes-Núñez, Silvia Salgado, Martha Sánchez, José Echevarría, Johanna Parrales, Andrés Tinizaray, Andrea Belén Salcedo Maldonado, Solón Alberto Orlando, Miguel Angel Garcia Bereguiain

**Affiliations:** 1 One Health Research Group, Universidad de Las Américas 126422, Quito, Ecuador; 2 Instituto Nacional de Salud Pública e Investigación 370452, Guayaquil, Ecuador; 3 Universidad Católica Santiago de Guayaquil 27887, Guayaquil, Ecuador; 4 Universidad ECOTEC 252896, Samborondón, Ecuador; National Chung Hsing University, Taichung, Taiwan; Wasit University, Wasit, Iraq; ICAR-National Institute of Veterinary Epidemiology and Disease Informatics, Bengaluru, Karnataka, India

**Keywords:** mpox, mpox virus, real-time PCR, molecular diagnosis, diagnostic performance, WHO, CDC

## Abstract

The global emergence and spread of monkeypox (mpox) have underscored the need for accurate and reliable molecular diagnostic tools. Real-time PCR remains the reference method for detecting mpox virus (MPXV); however, variations among available protocols may affect diagnostic performance. The objective of this study was to compare the diagnostic accuracy of the Centers for Disease Control and Prevention (CDC) of the USA real-time PCR assay with the World Health Organization (WHO) reference molecular protocol, using clinical samples from Ecuador. A retrospective analysis was conducted on 300 specimens from suspected mpox cases, including lesion swabs, vesicular samples, and serum, of which 150 were previously confirmed as MPXV-positive and 150 as MPXV-negative by the WHO assay. DNA was extracted using a standardized protocol, and all samples were tested with the CDC assay. The CDC protocol correctly identified 291 of 300 samples, yielding 141 true positives, 150 true negatives, 9 false negatives, and no false positives. The overall agreement was 97% (95% CI: 94–98), with a sensitivity of 94% (95% CI: 89–97) and a specificity of 100% (95% CI: 98–100). The positive predictive value was 100%, and the negative predictive value was 94% (95% CI: 90–97). The Cohen’s kappa coefficient obtained was 0.94 (95% CI: 0.90-0.98). These findings demonstrate that the CDC real-time PCR assay provides high diagnostic accuracy and excellent specificity for MPXV detection compared with the WHO reference protocol, supporting the use of both CDC and WHO real-time PCR assays as reliable diagnostic tools in clinical and surveillance settings.

**IMPORTANCE:**

The global emergence and spread of MPXV have underscored the need for accurate PCR diagnostic tools. This is the first study to address a comparison between the PCR protocols for MPXV from the Centers for Disease Control and Prevention (CDC) of the USA real-time PCR assay and the World Health Organization (WHO), two institutions globally acknowledged as references for infectious disease diagnosis. We show that both PCR protocols are very reliable for MPXV diagnosis and could be implemented for any laboratory worldwide indistinctly.

## INTRODUCTION

Mpox virus (MPXV) is an enveloped double-stranded DNA virus belonging to the *Orthopoxvirus* genus within the *Poxviridae* family. This virus exhibits greater environmental stability than most enveloped viruses, enabling persistence on contaminated surfaces, although it remains susceptible to common disinfectants, particularly organic solvents, likely due to its relatively low envelope lipid content (1–3). Phylogenetically, it is classified into two main clades: Clade I (previously the Congo Basin clade), associated with increased clinical severity, and Clade II (formerly the West African clade), characterized by comparatively lower virulence (4–6).

Mpox is a disease caused by MPXV, which continues to spread from endemic regions in Central and West Africa to countries in Europe and the Americas (7). The global outbreak that began in 2022 and was declared a Public Health Emergency of International Concern by the World Health Organization (WHO) was predominantly driven by the MPXV Clade II (subclade IIb) (7). This ongoing global outbreak has caused more than 100,000 cases in 122 total countries, including 115 countries where monkeypox was not previously reported (7). Moreover, there are ongoing outbreaks of Clade I MPXV in Central and Eastern Africa. Several countries in Western Europe began reporting subclade Ib cases since 2025 among individuals who had no documented history of international travel, while other travel-associated cases of clade I have been reported from other parts of Africa, as well as Asia, Europe, the Middle East, North America, South America, and Australia (7). Those travel-associated cases have almost exclusively been attributed to subclade Ib, with no deaths associated and mostly linked to relatively mild illness (7).

Mpox is a zoonotic infection transmitted through animal-to-human spillover and human-to-human spread, primarily via direct contact with skin or mucosal lesions, respiratory droplets, and sexual contact with infected individuals. Clinically, it is typically self-limiting and characterized by a skin rash, lymphadenopathy, fever, chills, headache, and other symptoms that resemble, but are generally milder than, those of smallpox (4, 7, 8).

The reference standard for confirming MPXV infection is real-time PCR (qPCR), owing to its high sensitivity and specificity in detecting viral DNA in clinical specimens, particularly from skin lesions, vesicular fluids, and scab samples (6–11). However, the effectiveness of molecular diagnosis may be compromised by the virus’s genetic variability (10). Reference international public health organizations have developed specific molecular protocols for MPXV detection. The WHO protocol, based on the work of Li et al. (11), is regarded as a worldwide gold standard for detecting unique viral DNA sequences by real-time PCR due to its high sensitivity and specificity. The molecular strategy is a multi-target approach comprising three independent assays using TaqMan hydrolysis probes to ensure diagnostic accuracy. The generic detection assay (*G2R_G*) targets the tumor necrosis factor receptor gene, enabling the identification of all MPXV strains, followed by clade differentiation using specific assays, including C3L for Clade I and G2R_WA for Clade II (12). The Centers for Disease Control and Prevention (CDC) of the USA has also implemented a real-time PCR assay targeting the tumor necrosis factor receptor gene. The CDC has reported significant deletions in this target region, which may compromise assay sensitivity for certain MPXV strains and increase the risk of false-negative results with the original primer and probe designs (13).

Despite the public availability of these protocols, independent scientific reports comparing their clinical performance in local settings remain limited. Validation of these diagnostic tools in regional diagnostic laboratories is essential to guarantee that MPXV strains of local or regional circulation remain detected. In this context, the aim of this study was to compare the diagnostic performance of the WHO and CDC real-time PCR protocols for detecting MPXV in clinical samples collected in Ecuador.

## MATERIALS AND METHODS

### Study design

This retrospective comparative study was performed using 300 residual clinical specimens from patients with suspected MPXV infection, including 229 skin lesion swabs, 36 vesicular lesion samples collected in Universal Transport Medium (UTM-RT, Copan Diagnostics, Italy), and 35 serum samples.

Among the total specimens, 150 had been previously confirmed as MPXV DNA positive, while the remaining 150 were negative or corresponded to other pathogens associated with similar clinical manifestations, including Epstein–Barr virus (EBV), varicella-zoster virus (VZV), and herpes simplex virus types 1 and 2 (HSV-1 and HSV-2). Samples were initially collected between May and December 2022 from patients of both sexes across a wide age range. Skin lesion swabs and vesicular lesion samples were obtained following standard clinical procedures and preserved in 3 mL of UTM-RT.

The performance of the CDC assay was qualitatively assessed by comparison with the WHO-recommended MPXV real-time PCR reference protocol, which served as the standard method for routine diagnostic confirmation. After initial testing, residual specimens were stored at −20°C until subsequent analysis.

### DNA extraction

Genomic DNA was isolated from leftover clinical specimens using the QIAamp DNA Mini Kit (QIAGEN, Hilden, Germany) according to the manufacturer’s protocol. In brief, 200 µL of each sample was subjected to extraction, and the nucleic acids were eluted in a final volume of 50 µL using the provided elution buffer.

### WHO reference molecular test

Real-time PCR reactions were performed in a final volume of 20 µL, consisting of 15 µL of master mix and 5 µL of extracted DNA, and included the human RNase P gene as an endogenous internal control (EIC) to verify sample integrity and exclude PCR inhibition. Positive control, extraction controls, and non-template (NTC) controls were also included. The real-time PCR thermal cycling for this protocol, as well as for the CDC assay, was carried out using the ABI 7500 Fast Real-Time PCR System (NWLP and Synnovis); the thermal conditions were initial incubation (50°C for 2 min), polymerase activation (95°C for 6–10 min), and 45 amplification cycles of denaturation (95°C for 15 s), followed by annealing/extension (60°C for 30 s for G2R_G and C3L, and 62°C for 30 s for G2R_WA), with fluorescence acquisition at each cycle. Results were interpreted based on cycle threshold (Ct) values: Ct ≤ 35 is considered positive; values between 36 and 40 are classified as weakly positive and should be interpreted with caution in the context of clinical and epidemiological data; and Ct values > 40 are considered negative (11, 12).

### CDC molecular assay

This assay incorporated essential controls to ensure analytical validity, including a positive MPXV control, a human DNA endogenous internal control (RNase P) to verify extraction efficiency and the absence of PCR inhibitors, an NTC to monitor potential contamination, and an extraction control based on human cellular material. Reactions were prepared in a final volume of 20 µL, consisting of 15 µL of master mix containing TaqMan primers and probes (FAM/BHQ-1) and 5 µL of template DNA. The thermal cycling profile included an initial denaturation step at 95°C for 20 s, followed by 40 amplification cycles of 95°C for 3 s and 60°C for 30 s, with fluorescence acquisition at each cycle. Results were interpreted according to Ct thresholds established during in-house validation; samples showing amplification with Ct < 40 were considered positive, while those without amplification or exceeding the established cutoff were considered negative (13).

### Statistical analysis

Statistical analyses were conducted using MedCalc software. The diagnostic performance of the CDC real-time PCR protocol was assessed against the WHO reference molecular assay. Performance metrics included clinical sensitivity (SE), specificity (SP), positive predictive value (PPV), negative predictive value (NPV), overall agreement (ORA), positive and negative likelihood ratios (LR + and LR−), Cohen’s kappa coefficient (κ), and McNemar’s Test (McNT), all calculated with 95% confidence intervals (95% CI). For comparative purposes, a confirmed MPXV case was defined as a specimen that tested positive on the WHO reference assay used in routine diagnostic workflows.

## RESULTS

A total of 300 clinical specimens were analyzed using both the WHO and the CDC real-time PCR protocols for MPXV detection. Additionally, a subset of samples tested negative for MPXV but were positive for other viral pathogens with similar clinical symptoms, including EBV (*n*=1), VZV (*n*=14), HSV-1 (*n*=5), and HSV-2 (*n*=4), were included in the study for a more accurate specificity analysis.

### Comparison of diagnostic performance between CDC and WHO MPXV real-time PCR protocols

Based on the WHO reference molecular assay, 150 specimens were confirmed positive for MPXV DNA, while the remaining 150 were negative.

The CDC assay correctly identified 291 out of the 300 specimens, yielding nine false-negative results and no false positives. The distribution of test outcomes is detailed in Table 1, and Table 2 presents the corresponding diagnostic performance metrics, demonstrating high accuracy for MPXV detection. Moreover, the Cohen’s kappa coefficient was 0.94 (95% CI: 0.90–0.98), indicating very good agreement between the CDC and WHO methods. Additionally, McNemar’s test revealed a statistically significant asymmetry between discordant pairs (exact *P* = 0.0039), with an absolute discordance rate of 3.0% (95% CI: 1.07–4.93), indicating that the CDC assay produced significantly more false-negative than false-positive results compared with the WHO assay.

**TABLE 1:** Confusion matrix of CDC MPXV real-time PCR assay versus the WHO reference protocol

		WHO protocol		
		(+)	(−)	Total
**CDC assay**	(+)	141	0	141
	(−)	9	150	159
	Total	150	150	300

**TABLE 2:** Diagnostic performance parameters of CDC assay compared with the WHO reference protocol for MPXV detection^*a*^

CDC assay vs WHO MPXV protocol (diagnostic performance measures)												
ORA	TP	TN	FP	FN	SE	SP	PPV	NPV	LR+	LR-	κ	McNT
0.97 (0.94–0.98)	141	150	0	9	0.94 (0.89–0.97)	1.00 (0.98–1.00)	1.00 (0.97–1.00)	0.94 (0.90–0.97)	∞ (not estimable)^*b*^	0.06 (0.03–0.11)	0.94 (0.90–0.98)	0.03 (0.01–0.05) *P* = 0.0039^*c*^

^*a*^ Overall agreement (ORA), true positive (TP), true negative (TN), false positive (FP), and false negative (FN), sensitivity (SE), specificity (SP), positive predictive value (PPV), negative predictive value (NPV), likelihood ratio positive (LR+), likelihood ratio negative (LR−), Cohen’s kappa coefficient (κ), McNemar’s Test (McNT), “*P”* value.

^*b*^ No false positives were observed, so the LR + is infinite (perfect discrimination among positives).

^*c*^ Absolute discordance rate. The 95% confidence intervals are shown in parentheses.

Seven of nine false-negative samples for the CDC protocol had Ct values greater than 36 (ranging from 36.2 to 38.7) in the WHO real-time PCR assay and should be considered weak-positive samples according to the reference method’s instructions. Only two of the nine false-negative samples for the CDC protocol had Ct values below 35 in the WHO assay (29.1 and 26.9). To rule out potential DNA degradation in these samples, the WHO MPXV real-time PCR assay was rerun on fresh DNA extractions prepared for the CDC real-time PCR protocol, and a positive result was confirmed for all nine samples. Nevertheless, if those weak positive samples are excluded as true positives in the comparison according to the WHO protocol cutoff of Ct < 35, the sensitivity of the CDC protocol would reach 98.6%.

## DISCUSSION

In recent years, global health systems have faced unprecedented challenges driven by the emergence and re-emergence of pathogens with epidemic and pandemic potential. This trend, exemplified by the SARS-CoV-2 crisis and, more recently, by the international spread of MPXV, highlights the vulnerability of healthcare systems to zoonotic diseases with high transmission potential (7). In this context, the availability of accurate and reliable molecular diagnostic tools has become essential for timely case identification and outbreak control (10, 11).

In the present study, the CDC MPXV real-time PCR protocol demonstrated high diagnostic accuracy, with an overall agreement of 97% and a Cohen’s kappa coefficient of 94%, compared with the WHO reference assay. McNemar’s exact test identified a statistically significant difference between paired results, exclusively attributable to nine false-negative results observed with the CDC assay, whereas no false-positive results were detected. These findings are consistent with previous reports indicating that PCR-based methods for MPXV detection exhibit excellent diagnostic performance. A recent meta-analysis reported pooled sensitivity and specificity estimates of 0.99 and 1.00, respectively, confirming PCR as a highly reliable tool for MPXV diagnosis (14). Similarly, multicenter evaluations have shown positive agreement rates above 95% even at lower viral loads, supporting the robustness of molecular assays across different laboratory settings (15).

The CDC assay evaluated in this study showed perfect specificity (100%) with no false-positive results. This indicates an excellent capacity to confirm MPXV infection, a critical feature in outbreak scenarios where false positives could lead to unnecessary public health interventions. Comparable studies have also reported high specificity and the absence of cross-reactivity with other pathogens, such as EBV, VZV, HSV-1, and HSV-2, which present similar clinical manifestations, thereby reinforcing the analytical reliability of MPXV real-time PCR-based assays (16).

The observed sensitivity was 94% for the CDC protocol compared with the WHO reference method. However, seven out of nine false-negative samples corresponded to weak-positive samples for the WHO reference method, which sets a cutoff of Ct < 35 for positivity, rather than Ct < 40 in the CDC protocol. So, to avoid bias in the comparison between these two protocols, if the seven weak-positive samples from the WHO protocol are excluded from the sensitivity calculation for the CDC protocol, the resulting value would be 98.6%. In the other hand, the two false-positive samples with Ct values around 30 for the WHO reference method could represent local circulating strains with genomic variability in the target regions that affect primer and probe binding in the CDC protocol. This is particularly relevant given that the CDC protocol targets specific genomic regions that may be subject to deletions or mutations, potentially affecting detection efficiency (15). Nevertheless, our results align with previous studies showing that PCR assay sensitivity can decrease significantly in samples with high Ct values, which are typically associated with low viral DNA concentrations (17).

Our study has an important limitation that we would like to acknowledge. All MPXV-positive samples included in this study belong to Clade II, the one responsible for the global outbreak initiated in 2022. Further studies are needed to compare the clinical performance of WHO and CDC real-time PCR protocols, including MPXV-positive samples from Clade I. During this study, no positive cases of Clade I were detected in Ecuador. In fact, the first and only case of MPXV Clade I in Ecuador was just reported in April 2026.

Overall, the findings of this study support either the use of the WHO or CDC real-time PCR protocols as highly accurate and reliable methods for MPXV detection in clinical specimens, including skin lesions and serum samples.

## Supplementary Material

Reviewer comments

## Data Availability

Data not included within the manuscript will be available upon request to the authors.

## SUPPLEMENTAL MATERIAL

OPEN PEER REVIEW reviewer-comments.pdf An accounting of the reviewer comments and feedback.

ASM does not own the copyrights to Supplemental Material that may be linked to, or accessed through, an article. The authors have granted ASM a non-exclusive, world-wide license to publish the Supplemental Material files. Please contact the corresponding author directly for reuse.
