# Supplementary material for: Comparison of diagnostic performance between the World Health Organization and the Centers for Disease Control and Prevention real-time PCR assays for molecular detection of the mpox virus
Source: Microbiol Spectr. 2026 Aug 14;14(9):e01431-26. doi: 10.1128/spectrum.01431-26 (PMC13532290; doi:10.1128/spectrum.01431-26)
Supplement: Supplementary file 1 [file reviewer-comments.pdf]

AMERICAN SOCIETY FOR MICROBIOLOGY | Microbiology Spectrum

Comparison of diagnostic performance between World Health Organization and Centers for Disease Control and Prevention of the USA real-time PCR assays for molecular detection of mpox virus

Darwin Paredes-Núñez, Silvia Paola Salgado Cisneros, Martha Sanchez, Jose Echevarría, Andres Tinizaray, Johanna Parrales, Andrea Belen Salcedo Maldonado, Solon Orlando, and Miguel Angel Garcia Bereguiain

Corresponding Author(s): Miguel Angel Garcia Bereguiain, Universidad de Las Americas

Review Timeline:

Submission Date:	April 27, 2026
Editorial Decision:	May 23, 2026
Revision Received:	June 6, 2026
Accepted:	June 21, 2026

Editor: Day-Yu Chao

Reviewer(s): Disclosure of reviewer identity is with reference to reviewer comments included in decision letter(s). The following individuals involved in review of your submission have agreed to reveal their identity: Hussein Mohammed Al.Bayati, H.A.M (Reviewer #1); Gundallahalli Bayyappa Manjunathareddy (Reviewer #2)

Transaction Report:

(Note: With the exception of the correction of typographical or spelling errors that could be a source of ambiguity, letters and reports are not edited. The original formatting of letters and referee reports may not be reflected in this compilation.)

DOI: https://doi.org/10.1128/spectrum.01431-26

Re: Spectrum01431-26 (**Comparison of diagnostic performance between World Health Organization and Centers for Disease Control and Prevention of the USA real-time PCR assays for molecular detection of mpox virus**)

Dear Dr. Johanna Parrales:

Thank you for the privilege of reviewing your work. Below you will find my comments, instructions from the Spectrum editorial office, and the reviewer comments.

Please return the manuscript within 60 days; if you cannot complete the modification within this time period, please contact me. If you do not wish to modify the manuscript and prefer to submit it to another journal, notify me immediately so that the manuscript may be formally withdrawn from consideration by Spectrum.

Revision Guidelines
To submit your modified manuscript, log into the submission site at https://spectrum.msubmit.net/cgi-bin/main.plex. Go to Author Tasks and click the appropriate manuscript title to begin. The information you entered when you first submitted the paper will be displayed; update this as necessary. Note the following requirements:

• Upload point-by-point responses to the issues raised by the reviewers in a file named "Response to Reviewers," NOT in your cover letter.
• Upload a compare copy of the manuscript (without figures) as a "Marked-Up Manuscript" file.
• Upload a clean .DOC/.DOCX version of the revised manuscript and remove the previous version.
• Each figure must be uploaded as a separate, editable, high-resolution file (TIFF or EPS preferred), and any multipanel figures must be assembled into one file.
• Any supplemental material intended for posting by ASM should be uploaded with their legends separate from the main manuscript. You can combine all supplemental material into one file (preferred) or split it into a maximum of 10 files with all associated legends included.

For complete guidelines on revision requirements, see our Submission and Review Process webpage. Submission of a paper that does not conform to guidelines may delay acceptance of your manuscript.

Data availability: ASM policy requires that data be available to the public upon online posting of the article, so please verify all links to sequence records, if present, and make sure that each number retrieves the full record of the data. If a new accession number is not linked or a link is broken, provide Spectrum production staff with the correct URL for the record. If the accession numbers for new data are not publicly accessible before the expected online posting of the article, publication may be delayed; please contact production staff (Spectrum@asmusa.org) immediately with the expected release date.

Publication Fees: For information on publication fees and which article types are subject to charges, visit our website. If your manuscript is accepted for publication and any fees apply, you will be contacted separately about payment during the production process; please follow the instructions in that e-mail. Arrangements for payment must be made before your article is published.

ASM Membership: Corresponding authors may join or renew ASM membership to obtain discounts on publication fees. Need to upgrade your membership level? Please contact Customer Service at Service@asmusa.org.

The ASM Journals program strives for constant improvement in our submission and publication process. Please tell us how we can improve your experience by taking this quick Author Survey.

Thank you for submitting your paper to Spectrum.

Sincerely,
Day-Yu Chao
Editor
Microbiology Spectrum

Reviewer #1 (Comments for the Author):

Hello friends
I would like to express my thankfulness and grateful for this invitation to this great job ,just need some modification in results

Reviewer #2 (Comments for the Author):

Comment 1: Cohen's Kappa Coefficient (κ)

Although sensitivity and specificity demonstrate the diagnostic performance of the CDC assay, they do not measure the overall agreement between the CDC and WHO assays beyond chance alone. Therefore, inclusion of Cohen's kappa coefficient (κ) would strengthen the statistical evaluation of assay concordance.
Including kappa analysis would provide stronger evidence that the CDC assay demonstrates excellent concordance with the WHO reference assay beyond random agreement.
Comment 2: McNemar's Test
McNemar's test is appropriate when two diagnostic methods are applied to the same set of samples and outcomes are categorical (positive/negative). Since both WHO and CDC real-time PCR assays were performed on the same 300 specimens, this statistical test is highly relevant.
McNemar's test evaluates whether the disagreement between the two assays is statistically significant. The test specifically focuses only on discordant pairs.
Since the number of discordant pairs is relatively low, the authors may additionally consider reporting the exact McNemar's test.
Comment 3: Ct Value Threshold Interpretation
The manuscript uses different positivity thresholds for the WHO and CDC assays:
This difference in Ct interpretation criteria may introduce bias when directly comparing diagnostic performance between the two assays.
Additionally, seven of the nine discordant samples had Ct values >36 according to the WHO assay, indicating low viral load or weak positive samples.
The authors should discuss more clearly how differing Ct cutoff criteria between the WHO and CDC protocols may influence sensitivity estimates and assay comparison.
Comment 4: Terminology Consistency for MPXV
The manuscript uses inconsistent terminology throughout the text, including:
• MPXV
• MPX
• MPVX
The abbreviation for monkeypox virus is MPXV, and terminology should be standardized consistently throughout the manuscript to improve scientific clarity and readability.
Comment 5: False Negative / False Positive Inconsistency
There appears to be an inconsistency in the Results section (line 182) regarding discordant samples.
The manuscript states:
"Only two of the nine false positive samples for the CDC protocol had Ct values below 35..."
However, according to Table 1 and the reported diagnostic performance:
• False positives (FP) = 0
• False negatives (FN) = 9
Therefore, the discordant samples described are actually false negatives, not false positives.
This statement should be corrected to avoid confusion in interpretation of assay performance.

References

1. https://www.statology.org/cohens-kappa-statistic/

2. Strassl, Robert, Karoline Rutter, Albert Friedrich Stättermayer, Sandra Beinhardt, Michael Kammer, Harald Hofer, Peter Ferenci, and Theresia Popow-Kraupp. "Real-time PCR assays for the quantification of HCV RNA: concordance, discrepancies and implications for response guided therapy." PloS one 10, no. 8 (2015): e0135963.

3. Mourtzikou, Antonia, Dimitrios Tsiftsis, Maria Agrogianni, Nikolaos Kasimatis, Anthoula Dritsa, Marilena Stamouli, Panagiotis Koumpouros, Christina Seitopoulou, and Elpida Toka. "A Synchronous Blind Test and Methodological Comparison Between Colloidal Gold Rapid Test vs RT-PCR For Sars-Cov-2 Detection." European Journal of Biomedical Research 1, no. 5 (2022): 7-12.

Dear Editor,

Please, see our point-by-point response to reviewers' comments.

We have also submitted the revised version of the manuscript with tracked changes in red for clarity, and a clean version as requested.

We believe we have addressed all the reviewers' comments and hopefully the manuscript is now suitable for publication.

Best regards

Miguel Angel Garcia-Bereguiain

Reviewer 1

I would like to express my thankfulness and grateful for this invitation to this great job, just need some modification in results.

Answer: Thanks for the positive feedback. We have improved the manuscript, including the results section as requested.

Reviewer 2

Comment 1: Cohen's Kappa Coefficient (κ)

Although sensitivity and specificity demonstrate the diagnostic performance of the CDC assay, they do not measure the overall agreement between the CDC and WHO assays beyond chance alone. Therefore, inclusion of Cohen's kappa coefficient (κ) would strengthen the statistical evaluation of assay concordance.

Including kappa analysis would provide stronger evidence that the CDC assay demonstrates excellent concordance with the WHO reference assay beyond random agreement.

Answer: Thanks for this great suggestion. Cohen's kappa coefficient has been calculated and included in the results section and also commented in the discussion.

Comment 2: McNemar's Test

McNemar's test is appropriate when two diagnostic methods are applied to the same set of samples and outcomes are categorical (positive/negative). Since both WHO and CDC real-time PCR assays were performed on the same 300 specimens, this statistical test is highly relevant.

McNemar's test evaluates whether the disagreement between the two assays is statistically significant.The test specifically focuses only on discordant pairs. Since the number of discordant pairs is relatively low, the authors may additionally consider reporting the exact McNemar's test.

Answer: Thanks again for the input. McNemar's Test has been run and included in the results section.

Comment 3: Ct Value Threshold Interpretation

The manuscript uses different positivity thresholds for the WHO and CDC assays: This difference in Ct interpretation criteria may introduce bias when directly comparing diagnostic performance between the two assays.
Additionally, seven of the nine discordant samples had Ct values >36 according to the WHO assay, indicating low viral load or weak positive samples.
The authors should discuss more clearly how differing Ct cutoff criteria between the WHO and CDC protocols may influence sensitivity estimates and assay comparison.

Answer: Thanks for pointing this out. We have now specifically mentioned the sensitivity value obtained for the CDC protocol depending on the cut off considered in the reference WHO protocol. This is stated both in the results and discussion sections.

Comment 4: Terminology Consistency for MPXV

The manuscript uses inconsistent terminology throughout the text, including: MPXV, MPX, MPVX. The abbreviation for monkeypox virus is MPXV, and terminology should be standardized consistently throughout the manuscript to improve scientific clarity and readability.

Answer: Sorry for this mistakes. It has been corrected now, and we use MPXV for monkeypox virus and mpox when we referred to monkeypox disease.

Comment 5: False Negative / False Positive Inconsistency

There appears to be an inconsistency in the Results section (line 182) regarding discordant samples.
The manuscript states: "Only two of the nine false positive samples for the CDC protocol had Ct values below 35..." However, according to Table 1 and the reported diagnostic performance: False positives (FP) = 0; False negatives (FN) = 9.
Therefore, the discordant samples described are actually false negatives, not false positives. This statement should be corrected to avoid confusion in interpretation of assay performance.

Answer: Sorry for this mistake. The discordant samples are indeed false negatives for CDC protocol. Thanks for catching this mistake. Corrected.

Re: Spectrum01431-26R1 (**Comparison of diagnostic performance between World Health Organization and Centers for Disease Control and Prevention of the USA real-time PCR assays for molecular detection of mpox virus**)

Dear Dr. Miguel Angel Garcia Bereguiain:

Your manuscript has been accepted, and I am forwarding it to the ASM production staff for publication. Your paper will first be checked to make sure all elements meet the technical requirements. ASM staff will contact you if anything needs to be revised before copyediting and production can begin. Otherwise, you will be notified when your proofs are ready to be viewed.

Data Availability: ASM policy requires that data be available to the public upon online posting of the article, so please verify all links to sequence records, if present, and make sure that each number retrieves the full record of the data. If a new accession number is not linked or a link is broken, provide production staff with the correct URL for the record. If the accession numbers for new data are not publicly accessible before the expected online posting of the article, publication may be delayed; please contact ASM production staff immediately with the expected release date.

Publication Fees: For information on publication fees and which article types have charges, please visit ourwebsite. We have partnered with Copyright Clearance Center (CCC) to collect author charges. If fees apply to your paper, you will receive a message from no-reply@copyright.com with further instructions. For questions related to paying charges through RightsLink, please contact CCC at ASM_Support@copyright.com or toll free at +1-877-622-5543. CCC makes every attempt to respond to all emails within 24 hours.

ASM Membership: Corresponding authors may join or renew ASM membership to obtain discounts on publication fees. Need to upgrade your membership level? Please contact Customer Service at Service@asmusa.org.

Embargo Policy: A press release may be issued as soon as the manuscript is posted on theSpectrum Latest Articles webpage. The corresponding author will receive an email with the subject line "ASM Journals Author Services Notification" when the article is available online.

The ASM Journals program strives for constant improvement in our submission and publication process. Please tell us how we can improve your experience by taking this quick Author Survey.

Thank you for submitting your paper to Spectrum.

Sincerely,
Day-Yu Chao
Editor
Microbiology Spectrum

Reviewer #1 (Comments for the Author):

Hello dear . i express my thankfulness for review and i expect it was sufficient for publication

Reviewer #2 (Comments for the Author):

Highlighting the changes or revisions made could have helped the reviewer to quickly glance the manuscript.
